# Colonization Dynamics of *Clostridioides difficile* in Suckling and Weaning Piglets

**DOI:** 10.3390/vetsci13050451

**Published:** 2026-05-03

**Authors:** Ana Martín Bermúdez, Eduardo Salido, Maria Jose Ramos-Real, Cintia Hernández-Sánchez, Maria Lecuona, Angeles Arias, Juan Carlos González, Carlos Beamonte, Miriam Hernández-Porto

**Affiliations:** 1 Department of Microbiology, Hospital Universitario de Canarias, Carretera Ofra s/n, 38320 San Cristóbal de La Laguna, Spain; amarberr@gobiernodecanarias.org (A.M.B.);; 2 Department of Pathology, Hospital Universitario de Canarias, Carretera Ofra s/n, 38320 San Cristóbal de La Laguna, Spain; 3 Department of Obstetrics, Gynecology, Pediatrics, Preventive Medicine and Public Health, Facultad de Ciencias de la Salud, Universidad de La Laguna, 38200 San Cristóbal de La Laguna, Spain; 4 Servicio Canario de Salud, Calle Pérez de Rozas 5, 38004 Santa Cruz de Tenerife, Spain; 5 General Directorate of Public Health, Calle Rambla de Santa Cruz 53, 38006 Santa Cruz de Tenerife, Spain

**Keywords:** *Clostridioides difficile*, ribotype 033 (RT033), zoonosis, One Health, binary toxin, toxin A, piglets

## Abstract

*C. difficile* is a major cause of antibiotic-associated diarrhea and hospital-acquired infections, although increasing community-acquired cases suggest alternative transmission routes. Livestock, particularly pigs, have been proposed as potential reservoirs. This study aimed to investigate the presence of zoonotic ribotypes in piglets from Tenerife (Spain) and to assess their pathogenic potential by detecting toxin genes. A total of 140 samples were analyzed, including 58 fecal samples from slaughtered piglets (4–8 weeks old) and 82 rectal swabs from piglets aged 2–25 days. Samples were cultured, identified by MALDI-TOF MS, and characterized by PCR ribotyping and toxin gene detection. No isolates were obtained from fecal samples collected at slaughter, whereas 14 (17%) rectal swabs were positive. Colonization was strongly age-dependent, with the highest prevalence at 2 days of age (100%), decreasing by day 9 (10.7%), and absent after 21 days (*p* < 0.05). All isolates were ribotype RT033 with a tcdA^+^/tcdB^−^/cdtA^+^/cdtB^+^ profile. The exclusive detection of RT033, a clade V lineage linked to animal reservoirs and occasional human infections, suggests a potential zoonotic risk, especially for farm workers. These findings reinforce the need for integrated *C. difficile* surveillance under a One Health framework to monitor emerging ribotypes and their role in community-acquired infections.

## Simple Summary

*Clostridioides difficile* is a bacterium that can cause diarrhea, especially after antibiotic use, and is commonly linked to hospitals. However, more infections are now being detected in the community, suggesting that other sources may exist. Farm animals, particularly pigs, have been proposed as possible carriers. In this study, we investigated whether young pigs from Tenerife (Spain) could harbor strains of this bacterium that might affect humans. We analyzed fecal and rectal samples from piglets of different ages. The bacterium was not found in piglets at slaughter, but it was detected in young piglets. Colonization was strongly age-dependent, peaking shortly after birth and disappearing within a few weeks. All detected strains belonged to ribotype 033 (clade V), and these strains carried genes linked to toxin production, albeit with an unusual pattern. These findings suggest that piglets may act as a temporary reservoir of this bacterium. Understanding this potential link between animals and humans is important for improving monitoring and prevention strategies from a One Health perspective, which considers the connections among human, animal, and environmental health.

## 1. Introduction

*C. difficile* is the leading cause of antibiotic-associated diarrhea and pseudomembranous colitis [1,2]. Furthermore, it represents one of the major causes of nosocomial infection in Europe. While traditionally considered a nosocomial pathogen, its increasing incidence in the community—estimated at 25% of cases—has highlighted the potential role of environmental and animal reservoirs [3,4, 5]. 

This microorganism is transmitted via the fecal–oral route via highly resistant spores. Upon ingestion, the spores surpass the gastric acid challenge and germinate in the intestine, successfully colonizing the colon. However, the manifestation of the disease is influenced by multiple concurrent factors, including comorbidities, medical treatments, patient age, and the state of their microbiota [6]. The pathogenic mechanism of this microorganism involves the release of toxins that directly affect the intestinal enterocyte, causing its lysis [7]. Furthermore, the persistence of spores in the environment through manure and agricultural waste, together with their high resistance to adverse conditions, confers great environmental persistence and favors their dissemination [8,9].

Although *Clostridioides difficile* is considered a major human pathogen, its pathogenic role in veterinary medicine remains a subject of debate due to the multifactorial nature of enteric processes in swine. While an association has been documented between diarrhea episodes in suckling and weaning piglets and the presence of strains exhibiting high toxin production—particularly the ST11/RT078 lineage—current evidence remains primarily circumstantial [10,11]. Given that other environmental factors and pathogens can influence the onset of clinical symptoms, it has not been definitively established whether *C. difficile* colonization inevitably leads to clinical disease.

One possible source of contagion is the consumption of and close contact with meat-producing animals. This is because *C. difficile* colonizes the intestinal tracts of multiple species, with prevalence varying by age and geographic region. Reported rates vary from 20% to 96% in piglets, 2% to 22% in cattle, and 6% to 15% in young goats [12,13,14,15,16,17]. Furthermore, spores can persist in the environment, particularly when manure and agricultural waste from colonized animals are used, thereby amplifying their dissemination in soils, water, and vegetables and contributing to greater human exposure even in the absence of direct animal contact [18].

Taken together, these findings suggest a possible zoonotic or environmental origin in the development of these community-acquired infections [12,19,20,21,22].

In the context of the One Health approach, the objectives of the present study were to determine the presence of emerging and zoonotic ribotypes in piglets that could represent a possible source of infection for humans in the community, and to evaluate their pathogenic potential by molecular detection of toxin genes.

## 2. Materials and Methods

### 2.1. Study Design and Population

This cross-sectional study evaluated the presence of *C. difficile* in piglets at different production stages. A total of 58 fecal samples were collected from piglets (4–8 weeks of age) at the Tenerife Island Slaughterhouse between December 2024 and February 2025. These animals originated from various independent commercial farms across the island. In parallel, 82 rectal swabs were obtained from piglets aged two to twenty-five days at a Yorkshire pig breeding farm in northern Tenerife. While the slaughterhouse sampling was conducted over several weeks, the farm sampling was restricted to a single-day visit due to strict biosecurity protocols, including the availability of all animals at that time.

The samples were collected from the piglets belonging to eight different breeding sows, with the following distribution: one sow contributed 8 piglets aged 2 days; five sows contributed 10, 10, 12, 12, and 12 piglets aged 9 days; one sow contributed 9 piglets aged 21 days; and another sow contributed 9 piglets aged 25 days. A rectal swab was obtained from each piglet. This selection allowed us to obtain a representative sampling of piglets of different ages for a more comprehensive analysis of *C. difficile* presence.

### 2.2. Sample Processing, Ribotyping, and Toxin Gene Detection

For the fecal samples, approximately 0.5 g of each sample was placed into microcentrifuge tubes (Eppendorf, Hamburg, Germany) containing 1 mL of 70% ethanol. Following a 20-min incubation at room temperature, the tubes were centrifuged at 4000 rpm for 10 min [23,24].

Rectal swabs were introduced into microcentrifuge tubes (Eppendorf) containing 1 mL of 70% ethanol and vortexed for 30 s. Subsequently, the tubes were incubated at room temperature for 20 min and then centrifuged at 4000 rpm for 10 min [23].

The resulting pellets from both sample types were inoculated onto chromogenic agar, specifically ChromID^®^ *C. difficile* Agar (bioMérieux, Marcy-l’Étoile, France). Plates were subsequently incubated at 36 ± 1 °C for 48 h under strict anaerobic conditions using anaerobic jars and AnaeroGen™ gas-generating sachets (Thermo Scientific, Waltham, MA, USA). The stability of the anaerobic atmosphere was continuously monitored using Oxoid™ Resazurin Anaerobic Indicators (Thermo Scientific) to verify that strictly anaerobic conditions were maintained throughout the incubation period. Identification of *C. difficile* was performed using the VITEK^®^ MS PRIME (bioMérieux, Marcy-l’Étoile, France) system, which is based on Matrix-Assisted Laser Desorption/Ionization–Time of Flight (MALDI-TOF) technology, for definitive identification.

**Figure 1:**
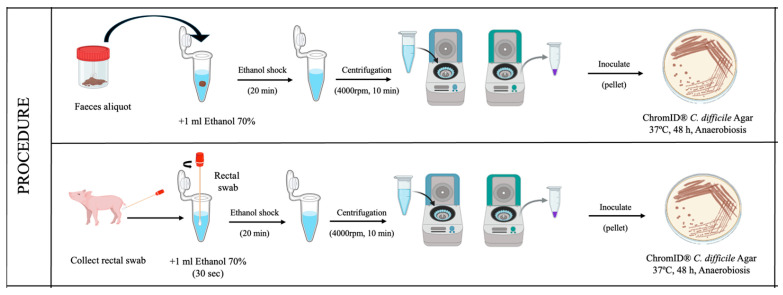
Schematic diagram of the sample processing procedure.

The complete sample collection and processing procedures for both fecal samples and rectal swabs are detailed in Figure 1.

Subsequently, each *C. difficile* isolate was cultured in thioglycolate broth under anaerobic conditions for 24 h.

The tubes were then centrifuged at 4000 rpm for 20 min. The resulting pellet was transferred into microcentrifuge tubes (Eppendorf, Hamburg, Germany) and centrifuged at 10,000 rpm for 10 min, and the supernatant was discarded. DNA extraction was performed using the QIAamp DNA Mini Kit (Qiagen, Hilden, Germany) according to the manufacturer’s instructions. The ribotyping of the *C. difficile* isolates was conducted following the ECDC’s Laboratory procedures for diagnosis and typing of human *Clostridium difficile* infection [25,26].

The amplification was performed using the primer set designed by Bidet et al. (FAM-5′-GTGCGGCTGGATCACCTCCT-3′ (16S) and 5′-CCCTGCACCCTTAATAACTTGACC-3′ (23S)). The 16S primers were labeled at the 5′ end with 6-carboxyfluorescein (6-FAM) [24]. Fragment analysis using capillary gel electrophoresis was carried out by the Genomics Service of the University of La Laguna using an AB3500 Analyzer (Applied Biosystems, Foster City, CA, USA), GeneScan 600 LIZ dye Size Standard v2.0, and POP-7 Polymer (Applied Biosystems, Foster City, CA, USA). Ribotype assignment was performed using the Webribo database (https://webribo.ages.at (accessed on 1 April 2025)) [27].

For the detection of toxin A gene (*tcdA*), toxin B gene (*tcdB*), and the binary toxin genes (*cdtA* and *cdtB*), individual PCRs were performed for each target and *C. difficile* isolate. These PCRs utilized the primers proposed by the ECDC [25,26].

For each PCR reaction, 1 μL of isolated DNA (10–100 ng), 0.5 μL of primer mix for each target (Table 1), and 15 μL of VWR^®^, Red Taq DNA Polymerase Master Mix were pipetted. The total reaction volume was 16.5 μL.

The PCR amplification program consisted of the following thermal cycling conditions: 94 °C for 3 min, (94 °C for 45 s, 50 °C for 45 s, 72 °C for 1 min) × 35 cycles, and a final 72 °C for 5 min.

A *C. difficile* RT078 reference strain, harboring all target genes, was used as a positive control, yielding peaks at 629 bp (*tcdA*), 410 bp (*tcdB*), 221 bp (*cdtA*), 262 bp (*cdtB*), and 158 bp (GDH). Nuclease-free water was used as a negative control.

### 2.3. Statistical Analyses

Data were analyzed using the IBM SPSS Statistics, version 22.0 for Windows (IBM Corp., Armonk, NY, USA). For pairwise comparisons of age groups on *C. difficile* prevalence, Fisher’s exact test was used. Furthermore, the Chi-square test for trend was used to assess the overall association between age and the presence of the pathogen. Prevalence estimates were reported with their corresponding 95% confidence intervals (95% CI) calculated using the Wilson Score method. In all cases, a *p*-value of less than 0.05 was considered statistically significant.

### 2.4. Ethical Considerations

The study was conducted in accordance with the Research Ethics and Animal Welfare Committee of the University of La Laguna (CEIBA2024-3464).

## 3. Results

### 3.1. Analysis of Fecal and Rectal Swab Samples

Fifty-eight fecal samples were analyzed from piglets aged between one and two months at the time of slaughter, yielding no *C. difficile* isolates.

On the other hand, analysis of 82 rectal swabs from piglets aged 2 to 25 days yielded 14 (17.1%) *C. difficile* isolates. Notably, pathogen detection was restricted to piglets aged 2 and 9 days (Table 2).

In the 2-day-old piglet group (9.76% of the total analyzed), all 8 samples were positive, yielding a 100% prevalence in this group. Notably, all positive samples within this cohort originated from a single sow. For the 9-day-old piglets, 56 samples (68.29% of the total) were analyzed, distributed across five litters corresponding to different sows. Six positive samples were detected, resulting in an overall prevalence of 10.71% in this age group. The distribution of cases was heterogeneous among litters: one litter had 3 positive piglets (25.0% of its litter), another had 2 (20.0%), and a third had 1 positive piglet (8.33%), while the remaining two litters yielded no isolates. Finally, no isolates were detected in the 21- and 25-day-old age groups (*n* = 18). This higher prevalence of *C. difficile* in the 2-day-old age group compared to other age groups was statistically significant (*p* < 0.05) (Table 3).

### 3.2. Ribotyping and Toxin Gene Detection

**Figure 2:**
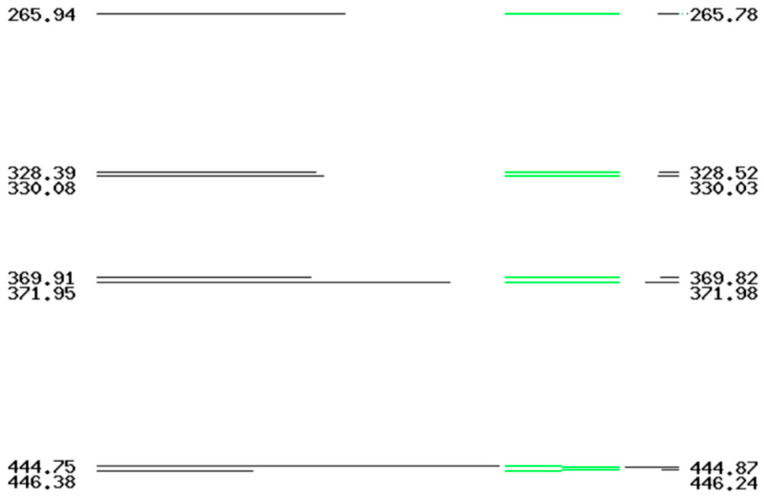
Comparison of the capillary electrophoresis ribotyping profile and PCR ribotyping profiles of *Clostridioides difficile* RT033. The figure displays the alignment between the ISR (Intergenic Spacer Region) peaks of a representative isolate from this study (left, black lines) and the RT033 reference profile from the database (right, green lines). Numbers indicate fragment sizes in base pairs (bp).

All isolates corresponded to ribotype RT033. The capillary gel-based electrophoresis ribotyping profiles of RT033 are shown in Figure 2.

**Figure 3:**
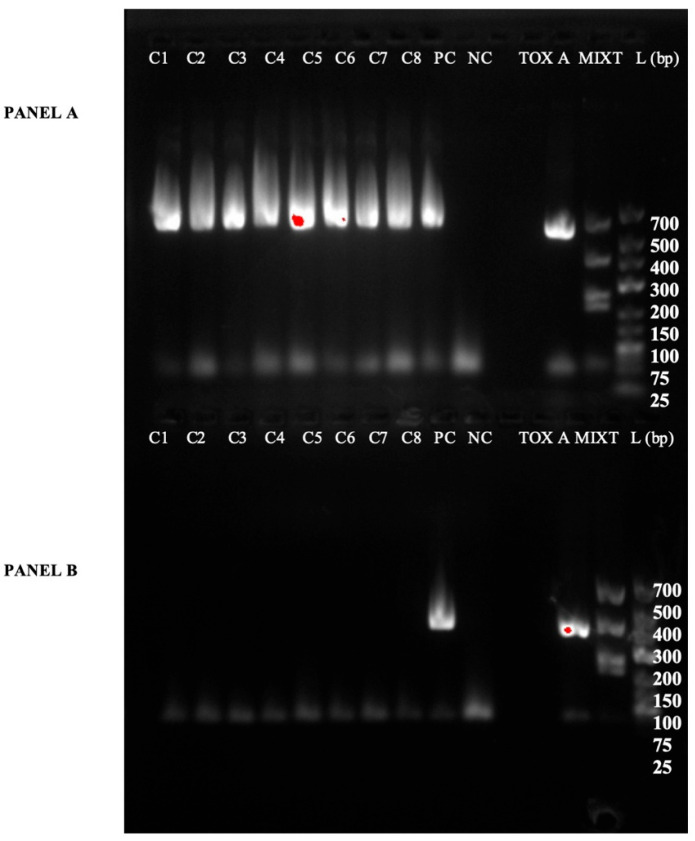
Gel electrophoresis analysis showing the PCR amplification results of toxin A and toxin B for eight *C. difficile* isolates obtained from piglets. The figure is divided into two parts (or panels): (**A**) shows the results of the toxin A (tcdA) PCR amplification. (**B**) shows the results of the toxin B (tcdB) PCR amplification. L: Molecular weight Ladder; PC: Positive Control for either the Toxin A or Toxin B PCR, as indicated by the respective panel; NC: Negative control; MIXT: Mix containing positive amplification products for Toxin A (*tcdA*), Toxin B (*tcdB*), and Binary Toxin (*cdtA* and *cdtB*); Lines 1–8 correspond to the eight different *C. difficile* isolates recovered from the piglets.

**Figure 4:**
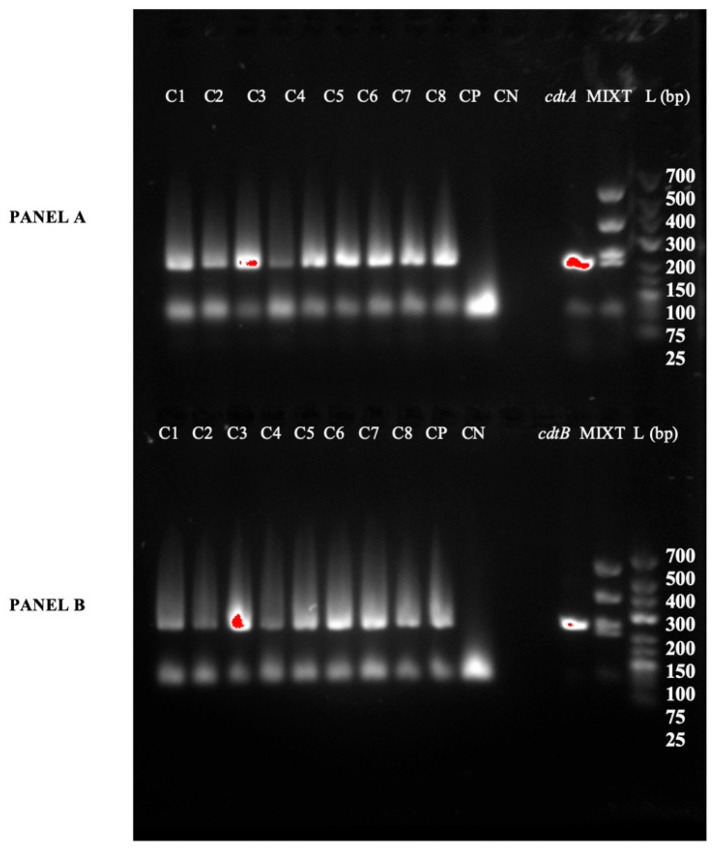
Gel electrophoresis analysis showing the amplification results of binary toxin A (*cdtA*) and binary toxin B (*cdtB*) PCRs for eight *C. difficile* isolates obtained from piglets. The figure is divided into two parts (or panels): (**A**) shows the results of the cdtA PCR amplification. (**B**) shows the results of the cdtB PCR amplification. L: Molecular weight Ladder; CP: Positive Control for either the *cdtA* or *cdtB* PCR, as indicated by the respective panel; CN: Negative control; MIXT: Mix containing positive amplification products for Toxin A (*tcdA*), Toxin B (*tcdB*), and Binary Toxin (*cdtA* and *cdtB*); Lanes 1–8 correspond to the eight different *C. difficile* isolates recovered from the piglets.

Furthermore, all isolates carried the genes encoding toxin A and binary toxin (cdtA/cdtB). However, the *tcdB* gene was not detected (Figure 3 and Figure 4).

## 4. Discussion

The colonization by *C. difficile* in piglets appeared to follow a transient pattern over time under the conditions of this study. In our study, a prevalence of 100% was observed during the first days of life, which sharply decreased to 10.71% by 9 days of age and became null starting from the third week; this was consistent across piglets from the breeding farm (21–25 days of age) and those sampled at the time of slaughter (1–2 months of age). Although no statistically significant differences were observed between the 9-day and 21–25-day groups (*p* = 0.325), a decreasing trend in *C. difficile* detection with increasing age was noted, consistent with its known epidemiology in piglets. However, a post hoc power analysis (23.8%) indicated that the limited sample size likely constrained the ability to detect significant differences; therefore, these findings should be interpreted with caution. Further studies with a larger sample size are necessary to confirm these observations and provide sufficient statistical power to validate this age-dependent colonization pattern.

The high colonization rate during the first days of life is attributed to multiple factors, including an immature and poorly diversified intestinal microbiota, resulting in scant bacterial competition that allows the proliferation of opportunistic microorganisms such as *C. difficile*, the absence or low level of secondary bile acids, which inhibit *C. difficile* spore germination, and a developing immune system, which is still incapable of effectively limiting colonization [17,28,29,30].

Similarly, Usui et al. documented prevalences of up to 57.5% in piglets compared to 0.8% in adult pigs, reinforcing the role of young animals as reservoirs [31]. This progressive reduction in postnatal colonization is driven by the rapid increase in intestinal microbiota diversity from the second week onward, which favors the displacement of *C. difficile* through bacterial competition and prevents new colonization [28,29]. The disappearance of the microorganism can be interpreted as a physiological marker of the transition toward a more resilient microbiome against spore-forming pathogens [28,32]. Nutritional factors, such as weaning and the introduction of solid foods, are closely associated with structural changes in the intestinal ecosystem that favor greater microbial stability and enhanced defense capacity against colonization by enteric pathogens [6,17,32]. On the other hand, the absence of *C. difficile* at the time of slaughter in our study is consistent with the findings of Doyle et al. and Candel et al., who also did not detect the bacterium in porcine meat [33,34]. While our results align with these findings, it is noteworthy that other studies have reported its low prevalence in porcine meat [35].

In our findings, all isolates corresponded to *C. difficile* ribotype 033 (RT033), which falls within *C. difficile* clade V. This clade is genetically related to other ribotypes such as 078 and 126. According to the literature, it is the one most linked to the animal reservoir and zoonotic transmission, primarily in calves and piglets [36,37,38,39,40,41]. While RT033 has been isolated from symptomatic patients, its role in human disease is often considered rare and remains poorly understood [42,43]. This is largely because RT033 typically lacks the classical toxins A and B, producing only the binary toxin (CDT) [44].

Traditionally, toxins A (*tcdA*, enterotoxin) and B (*tcdB*, cytotoxin), encoded in the Pathogenicity Locus (PaLoc), have been viewed as the primary drivers of *C. difficile* virulence [3,7,24,45]. However, the role of the binary toxin (CDT)—encoded in the independent *CdtLoc*—is gaining recognition. Although its exact pathogenic role is not yet fully elucidated, the presence of CDT has been associated with increased virulence and bacterial adherence capacity [10]. Furthermore, Meza-Torres et al. demonstrated that CDT not only exacerbates acute virulence but also promotes the persistence and recurrence of infection [46].

In our study, the presence of a specific fragment of the *tcdA* gene was detected, resulting in a *tcdA^+^/tcdB^−^/cdtA^+^/cdtB^+^* genetic profile. This finding is infrequent but aligns with observations by Spigaglia et al., who reported a similar pattern in 2.6% of livestock isolates [16]. It is important to note that the detection of Toxin A genes by standard PCR (such as the ECDC-recommended assays) does not always guarantee a functional Toxin A. In many RT033 strains, the PaLoc harbors significant deletions that affect the amplified region, potentially leading to nonfunctional gene fragments [45,47].

Conversely, some studies have documented strains with a truly intact gene in the absence of tcdB [48,49]. Characterization using whole-genome sequencing confirmed that the *tcdA* gene was present at its full length, with no evidence of Toxin B. Research by Monot et al. highlights that the PaLoc is a highly dynamic region that can be gained or lost through homologous recombination between toxigenic and non-toxigenic strains. This genomic plasticity generates intermediate configurations and atypical profiles that maintain pathogenic potential despite the absence of one of the major toxins [48,49,50].

On the other hand, *C. difficile* exhibits high environmental dispersion and elevated direct transmissibility to humans in livestock settings, particularly in pig and cattle farming [9,22,51,52]. This high transmissibility may facilitate the introduction of livestock lineages, such as RT033, into the community. Consequently, this ribotype should be proactively sought in the community; current clinical search protocols may be insufficient since these populations do not present the typical risk factors. In contrast to nosocomial infections, community-acquired infections typically occur in younger patients (<65 years) without classic risk factors or recent antibiotic exposure, and the exact source of infection is often unknown [53,54].

In this context, the pathogenicity of ribotype 033 in humans may be underestimated, as this lineage typically lacks toxins A and B, making it invisible to commercial enzymatic assays that exclusively detect those two toxins [43,44,47,55].

From a public health perspective, the presence of RT033 in livestock represents a significant challenge for human surveillance. Most current clinical diagnostic guidelines for *C. difficile* infection focus primarily on the detection of toxins A and B [56,57,58]. Consequently, if these zoonotic binary toxin-positive strains were to jump to humans, they could be systematically underdiagnosed. This underscores the need to integrate binary toxin detection into clinical algorithms, particularly in cases where other enteric pathogens have been ruled out, to prevent the silent dissemination of emergent lineages from animal reservoirs.

Several limitations should be acknowledged. First, a comparative analysis with community-acquired human cases was not feasible, as routine diagnostic screening for *C. difficile* in patients with diarrhea is not standard practice in our region, precluding direct comparison with clinical data. Second, within the animal production chain, exact figures for piglets originating from the breeding farm at the time of slaughter were unavailable, and traceability at the slaughterhouse did not allow identification of the farms of origin.

Methodologically, logistical constraints limited the sample size, and strict biosecurity protocols restricted the farm sampling to a single-day visit, resulting in single-litter representation for most age groups. Given the limited sampling depth, our results should be regarded as exploratory. Consequently, while these findings provide valuable insights into colonization trends, they should be interpreted with caution as they may not fully capture the maternal and genetic variability of the entire population. Furthermore, as this was a cross-sectional study, individual piglets were not tracked longitudinally from birth to slaughter. Therefore, the observed decline represents a snapshot of age-related prevalence at the population level rather than a confirmed individual clearance. In addition, rectal swabs were used instead of fecal samples, which are generally more sensitive; therefore, prevalence in neonates may be underestimated. However, the detection of *C. difficile* in younger piglets but not in older animals supports the observed decrease in colonization with age. The lack of maternal screening (sows) and livestock personnel testing prevents a definitive assessment of the transmission pathways or a direct molecular comparison to confirm zoonotic linkage in this specific setting.

Finally, detection relied exclusively on culture-based methods, which may have lower sensitivity than direct PCR in low-bacterial-load samples, despite the use of alcohol shock to enhance spore recovery. Whole-genome sequencing (cgMLST and SNP analysis) was not performed due to budget constraints, limiting comparison of the RT033 strain with international databases and human isolates. Moreover, although a fragment of the *tcdA* gene was detected, its full integrity and functional expression were not confirmed.

## 5. Conclusions

In summary, our findings suggest a clear age-dependent colonization pattern of *C. difficile* in piglets, where RT033—a ribotype closely related to Clade V lineages like 078 and 126—acts as a transient colonizer restricted to the first weeks of life. While this study is limited by the absence of human, sow, and farm worker sampling, as well as WGS-based linkage analysis, these observations underscore the importance of maintaining integrated surveillance under the One Health approach. Such monitoring may provide essential insights into transmission dynamics and help limit the potential dissemination of emergent lineages.

## Figures and Tables

**Table 1:** Sequence primer and concentration.

Name	Primer Sequence	Primer Concentration	Target Gene
*GluD-F*	(5′-GTC TTGGATGGTTGATGAGTAC-3′)	25 μM	GDH
*GluD-R*	(5′-TTCCTAATTTAGCAGCAGCTTC-3′)	25 μM	
*tcdA-F*	(5′-GCATGATAAGGCAACTTCAGTGGTA-3′)	25 μM	Toxin A
*tcdA-R*	(5′-AGTTCCTCCTGCTCCATCAAATG-3′)	25 μM	
*tcdB-F*	(5′-CCAAARTGGAGTGTTACAAACAGGTG-3′)	25 μM	Toxin B
*tcdB-RA*	(5′-GCATTTCTCCATTCTCAGCAAAGTA-3′)	12.5 μM	
*tcdB-RB*	(5′-GCATTTCTCCGTTTTCAGCAAAGTA-3′).	12.5 μM	
*cdtA-FA*	(5′-GGGAAGCACTATATTAAAGCAGAAGC-3′)	12.5 μM	Toxin cdtA
*cdtA-FB*	(5′-GGGAAACATTATATTAAAGCAGAAGC-3′)	12.5 μM	
*cdtA-R*	(5′-CTGGGTTAGGATTATTTACTGGACCA-3′)	25 μM	
*cdtB-F*	(5′-TTGACCCAAAGTTGATGTCTGATTG-3′)	25 μM	Toxin cdtB
*cdtB-R*	(5′-CGGATCTCTTGCTTCAGTCTTTATAG-3′)	25 μM	

**Table 2:** Distribution of analyzed rectal swab samples.

Sow ID	Piglet Age (Days)	Number of Piglets (% of Total)	*C. difficile*-Colonized Piglets (%)	95% CI
Sow 1	2	8 (9.8)	8 (100.0)	(67.6–100.0)
Sow 2	9	10 (12.2)	0 (0)	(0.0–28.3)
Sow 3	9	12 (14.6)	3 (25)	(8.9–53.2)
Sow 4	9	10 (12.2)	2 (20)	(5.7–51.0)
Sow 5	9	12 (14.6)	1 (8.3)	(1.5–35.4)
Sow 6	9	12 (14.6)	0 (0)	(0.0–24.2)
Sow 7	21	9 (11)	0 (0)	(0.0–30.8)
Sow 8	25	9 (11)	0 (0)	(0.0–30.8)
Total	—	82 (100)	14 (17)	(10.5–26.5)

**Table 3:** Pairwise Comparison of *C. difficile* Prevalence in Piglets by Age Group.

Comparison (Group A vs. Group B)	Group A (Positive/Negative)	Group B (Positive/Negative)	*p*-Value Fisher’s Exact Test
2 days vs. 9 days	8/0	6/50	<0.000001
2 days vs. 21–25 days	8/0	0/18	<0.000001
2 days vs. >30 days	8/0	0/58	<0.000001
9 days vs. 21–25 days	6/50	0/18	0.325
9 days vs. >30 days *	6/50	0/58	0.012
21–25 days vs. >30 days *	0/18	0/58	1.000

Data presented show the absolute counts of positive and negative *C. difficile* isolations for each age group comparison. * >30 days’ group consists of the 58 samples collected at the slaughterhouse (animals aged 1–2 months). Statistical significance was determined using Fisher’s exact test, with a significance threshold of *p* < 0.05.

## Data Availability

The original contributions presented in this study are included in the article Further inquiries can be directed to the corresponding author.
